# Evaluating Polymer Characterization Methods to Establish a Quantitative Method of Compositional Analysis Using a Polyvinyl Alcohol (PVA)/Polyethylene Glycol (PEG)—Based Hydrogel for Biomedical Applications

**DOI:** 10.3390/polym18010048

**Published:** 2025-12-24

**Authors:** Antonio G. Abbondandolo, Anthony Lowman, Erik C. Brewer

**Affiliations:** Department of Biomedical Engineering, Rowan University, Glassboro, NJ 08028, USA

**Keywords:** polyvinyl alcohol (PVA), polyethylene glycol (PEG), hydrogel, freeze-thaw

## Abstract

Multi-component polymer hydrogels present complex physiochemical interactions that make accurate compositional analysis challenging. This study evaluates three analytical techniques: Nuclear Magnetic Resonance (NMR), Advanced Polymer Chromatography (APC), and Thermogravimetric Analysis (TGA) to quantify polyvinyl alcohol (PVA) and polyethylene glycol (PEG) content in hybrid freeze-thaw derived PVA/PEG/PVP hydrogels. Hydrogels were synthesized using an adapted freeze–thaw method across a wide range of PVA:PEG ratios, with PVP included at 1 wt% to assess potential intermolecular effects. NMR and APC reliably quantified polymer content with low average errors of 2.77% and 2.01%, respectively, and were unaffected by phase separation or hydrogen bonding within the composite matrix. TGA enabled accurate quantification at PVA contents ≤ 62.5%, where PEG and PVA maintained distinct thermal decomposition behaviors. At higher PVA concentrations, increased hydrogen bonding and crystalline restructuring, confirmed by FTIR through shifts near 1140 cm^−1^ and significant changes in the -OH region, altered thermal profiles and reduced TGA accuracy. Together, these findings establish APC as a high-throughput alternative to NMR for multi-component polymer analysis and outline critical thermal and structural thresholds that influence TGA-based quantification. This work provides a framework for characterizing complex polymer networks in biomedical hydrogel systems.

## 1. Introduction

Variations in intermolecular interactions within polymeric materials directly affect their resulting chemical and physical properties. Polyvinyl alcohol (PVA) is a synthetic polymer that is increasingly being used in biomedical applications due to its excellent biocompatibility, aqueous solubility, low toxicity, and tissue-mimicking properties [1]. In the biomedical space, it has been utilized in contact lenses [2], artificial pancreases [3], hemodialysis [4], tissue adhesion barriers [5], cartilage [6,7,8] and meniscus tissue replacements [9], wound dressings [10], degradable drug delivery systems [11], and as a nucleus pulposus replacement [12]. PVA can be manufactured with varying degrees of hydrolysis and molecular weight, resulting in an overall change on its chemical and physical properties and crystallinity behavior, allowing for fine-tuning of physiochemical properties depending on the desired application [1]. Whereas the properties of single polymer systems are generally well-understood, physiochemical insight into multi-component polymer networks is poorly defined.

The properties of each individual polymer contribute to the overall physiochemical nature of the composite. Physical crosslinking is a more desirable, non-toxic method of hydrogel formulation that allows for the modification of physiochemical properties of PVA through hydrogen bonding with other polymers [1]. Thomas et al. [13] previously developed a polyvinyl alcohol (PVA)/polyvinylpyrrolidone (PVP) hydrogel for nucleus pulposus replacement and demonstrated that hydrogel swelling was strongly dependent on the PVA/PVP ratio. Specifically, formulations with higher PVP content exhibited increased swelling, which was attributed to the amorphous structure of PVP and its greater affinity for water. This effect was further amplified with increasing molecular weights of both PVA and PVP [13]. Conversely, Liu et al. [14] developed a physically crosslinked PVA/Gelatin hydrogel and described identical thermal melting properties between hydrogels produced through PVA alone and hybrid materials. The authors attributed the similar thermal properties to the weak hydrogen bonding between polymers, minimally influencing the thermal characteristics of the hydrogel.

A less common method for producing PVA hydrogels is a hybrid approach developed by Ruberti and Braithwaite [15]. In this technique, a gelling agent such as low molecular weight Polyethylene glycol (PEG) decreases the solvent quality (e.g., water) in the PVA solution. This causes phase separation in the PVA, forming polymer-rich and solvent-rich regions, with the PVA crystallizing in the polymer-rich areas without the need for freeze-thaw cycles [16]. The addition of PEG to the aqueous PVA solution leads to phase separation, creating a polymer-dense gel phase and a dilute, water-dense supernatant when PEG with a molecular weight above 600 Da is used. Lim [17] observed a reduction in hydrogen bonding in vinyl alcohol units caused by PEG, which was identified using FTIR analysis. Furthermore, Lim found that the molecular weight of PEG influences its plasticizing effect [17] and impacts crystallite formation in PVA films (87% hydrolyzed), suggesting that PEG introduces defects into the PVA crystal lattice.

As phase-separated hydrogels present a multi-component system consisting of a polymer-rich hydrogel and aqueous-based supernatant, it is challenging to extrapolate molecular behavior of individual components to the entire characteristics and nature of the composite polymer matrix [18]. Thus, it is necessary to use multiple methods of polymer analysis to determine the physiochemical and material properties of a multi-component polymer matrix, such as a PVA/PEG/PVP hydrogel for biomedical applications. Furthermore, as these hydrogels present two unique phase separations, it is important to understand the polymer composition of each component during manufacturing. Typically, the composition of multicomponent polymer systems is characterized by ^1^H Nuclear Magnetic Resonance (NMR) Spectroscopy [19,20]. It is particularly useful for polymer chemical structure and compositional analysis as nearly all polymers have unique protons for analysis. Gel permeation chromatography (GPC) and other similar size exclusion methods such as APC can be used for compositional analysis of blends [21,22] by separating polymers according to their respective size and molecular weight, but are oftentimes challenging due to overlapping peaks, requiring the need for peak deconvolution or use of another analysis method [23]. In this work, we propose a novel method of individual polymer composition analysis using Advanced Polymer Chromatography (APC) and Thermal Gravimetric Analysis (TGA). Nuclear Magnetic Resonance (NMR) will be used to validate the proposed method and functional group interactions between polymer components will be identified and compared using Fourier Transform Infrared Spectroscopy (FTIR) to better understand physiochemical changes with composite hydrogels with varying polymer concentrations.

## 2. Materials and Methods

### 2.1. Materials

PVA with a molecular weight of 145,000 g∙mol^−1^ and >99% hydrolyzed was obtained from EMD Millipore Corporation (Billerica, MA, USA). PEG with a molecular weight of 1000 g∙mol^−1^ and PVP with a molecular weight of 40,000 g∙mol^−1^ were obtained from Spectrum Chemical (New Brunswick, NJ, USA). Deionized (DI) water was obtained on-site using a Millipore Milli-Q system (Darmstadt, Germany). For the APC eluent, sodium nitrate (NaNO_3_) was obtained from MilliporeSigma (Burlington, MA, USA) and HPLC grade, 99.9% dimethyl sulfoxide (DMSO) was purchased from Thermo Fisher Scientific (Waltham, MA, USA). Deuterium oxide (D_2_O) was purchased from TCI America (Portland, OR, USA) for NMR analysis.

### 2.2. Hydrogel Preparation

Hydrogels were prepared using an adapted freeze-thaw method previously described by Peppas [24] and LaMastro [25]. Briefly, aqueous solutions of PVA were prepared by steam autoclaving sealed bottles containing the previously mentioned raw materials at 121 °C at 30 psi for 30 min. Within 10 min after the first autoclave cycle, the solution was stirred manually by spatula to ensure complete dissolution of the two polymers. While the solutions were still hot, PEG and additional DI water was then added to sterile glass vials to make several different formulations summarized in Table 1. The solutions were then stirred manually again and placed in a 100 °C thermal heating oven for 2 h to ensure complete dissolution of polymer components. The solutions were then cooled at room temperature for 3 h and then placed in a −20 °C freezer overnight to initiate the cryo-gel formation. All samples were subject to one freeze-thaw cycle to induce hydrogel formation. The total mass of components was fixed at 10 g to ensure sufficient material for all testing procedures. Hydrogels were made with and without PVP to assess the potential intermolecular changes that a small addition of the polymer may initiate. Hydrogels made with PVP were prepared by autoclaving aqueous solutions of PVA and PVP (99:1 *w*/*w*%) following the same procedure outlined previously. PVP content was assumed negligible for subsequent quantitative analysis due to its minimal contribution to the total mass of the biomaterial and overlapping spectral and thermal properties with PVA.

### 2.3. Polymer Compositional Analysis

To understand the final hydrogel composition following phase separations, samples were lyophilized at −80 °C for at least 72 h with a Labconco FreeZone 4.5 L Benchtop Freeze Dry System. Samples were then cryogenically milled using a RETSCH cryomill (Haan, Germany) and liquid nitrogen feed supply to maximize polymer surface area and ensure polymer homogenization during further testing. The hydrogels were individually ground for a total of 5 cycles with a pre-cooling time of 1:30 min at 5 Hz, grinding time of 3:30 min at 30 Hz, and intermediate cooling time of 1:00 min at 5 Hz.

#### 2.3.1. NMR

10 mg of each ground hydrogel formulation was weighed and placed into sterile glass vials with 1 mL of deuterium oxide (D_2_O). Samples were heated at 100 °C for at least 1 h to allow for complete dissolution before being pipetted to NMR testing tubes and subsequently analyzed. ^1^H NMR spectra were recorded on a Fourier transform nuclear magnetic resonance spectrometer (Bruker AV II-400 MHz; Bruker Corporation, Fällanden, Switzerland) at 25 °C.

#### 2.3.2. APC

Briefly, a 0.01M sodium nitrate (NaNO_3_) solution of 95/5 *v*/*v* % DI water and dimethyl sulfoxide (DMSO) was made to dissolve test articles and as the eluent in the mobile phase. A small amount of sample was weighed in a sterile glass vial and dissolved in enough mobile phase so that the concentration of the sample was 4 mg/mL. The glass vial was then placed on a hot plate at 100 °C for 1 h to allow for complete dissolution of the polymer. After complete dissolution, the sample was filtered through a 0.1 µm PTFE syringe filter and transferred to a Waters^®^ Clear Glass 12 × 32 mm Screw Neck Vial with PTFE/Silicone Preslit Septum.

An ACQUITY APC AQ BEH Column, 200 Å, 2.5 µm, 4.6 mm × 75 mm and ACQUITY APC AQ BEH Column, 125 Å, 2.5 µm, 4.6 mm × 30 mm were connected in series with an XBridge Protein BEH SEC Guard Column, 125 Å, 2.5 µm, 4.6 mm × 30 mm during testing. Samples were tested at a flow rate of 0.3 mL/min and an injection volume of 10 µL was used. The column and refractive index (RI) temperature were set to 50 °C on the Waters^®^ Acquity APC System. The RI was purged with methanol for 70 min and the mobile phase was allowed to equilibrate for 30 min before subsequent testing.

To allow for individual quantification of PVA and PEG within the hydrogel composite, concentration curves for each polymer were made by making respective samples of 4, 3, 2, 1, 0.5, and 0.25 mg/mL. The area under the curve of each polymer was recorded and used to create a linear relationship between concentration and mV, where the slope of the model was used for theoretical concentration calculations. The concentration curves for PVA and PEG, respectively, can be found in Figures S1 and S2.

#### 2.3.3. PVA/PEG Hydrogel Component Analysis Through the Use of TGA

100 µL platinum TGA sample pans were cleaned and tared before use. All TGA testing was conducted in air with a sample flow rate of 25 mL/min and balance flow of 40 mL/min. Preliminary testing of individual polymer materials of the hydrogel indicated complete decomposition of PEG and PVA at 250 °C and 550 °C, respectively (see Figures S3 and S4). Minimal weight loss was identified for PVA until 300 °C and the combination of both materials’ distinct thermal patterns, identified by plateaus for each corresponding polymer material (see Figure S5), allows for development of a TGA method to quantify polymer content. The developed method applied a 10 °C/min temperature ramp until 250 °C after equilibration at 30 °C, followed by 60 min of isothermal heating. A secondary temperature ramp was applied until 550 °C and the temperature was held isothermally for an additional 45 min. Finally, a third temperature ramp was applied until 800 °C.

Mass % calculations were obtained by subtracting weight percent values throughout isothermal holding cycles to get a representative indication of PEG and PVA content. For all calculations, the *weight%* of PVP was assumed negligible due to its minimal mass contribution to the overall components of the biomaterial. In order to calculate the weight percent of PEG in the sample, the initial weight percent at the start of the experiment was subtracted from the value at the end of the 250 °C heating cycle (Equation (1)).

(1) $$PEG\;Weight\;\%=Weight{\%}_{Starting\;}-Weight{\%}_{End250\;{}^{\circ}C}\;$$

To calculate the weight percent of PVA in the sample, the initial weight percent at the start of the experiment was subtracted from the value at the end of the 550 °C isothermal heating cycle. This value is representative of the total PEG and PVA content in the whole sample. To calculate the PVA content, the previously found value for PEG weight % was subtracted from the total PEG and PVA content (Equation (2)).

(2) $$PVA\;Weight\;\%=Weight{\%}_{Starting\;}-Weight{\%}_{End550\;{}^{\circ}C}-Weight{\%}_{PEG}$$

#### 2.3.4. FTIR

FTIR spectra were collected using a Nicolet is50 Spectrometer (ThermoFischer Scientific, Waltham, MA, USA) equipped with a KBr beam splitter, and single reflection diamond Attenuated Total Reflectance (ATR) attachment. Spectra were obtained with 32 scans and 4 cm^−1^ resolution in the range of 4000 cm^−1^ to 650 cm^−1^. Spectra were preprocessed and normalized using the Omnic^TM^ software suite v9.1 from ThermoFischer Scientific, Waltham, MA, USA. A modified specific area under band (SAUB) method from Wallis et al. [26] was used to calculate the area of the -OH bonding region between 3650 cm^−1^–3000 cm^−1^.

## 3. Results

### 3.1. NMR

**Figure 1:**
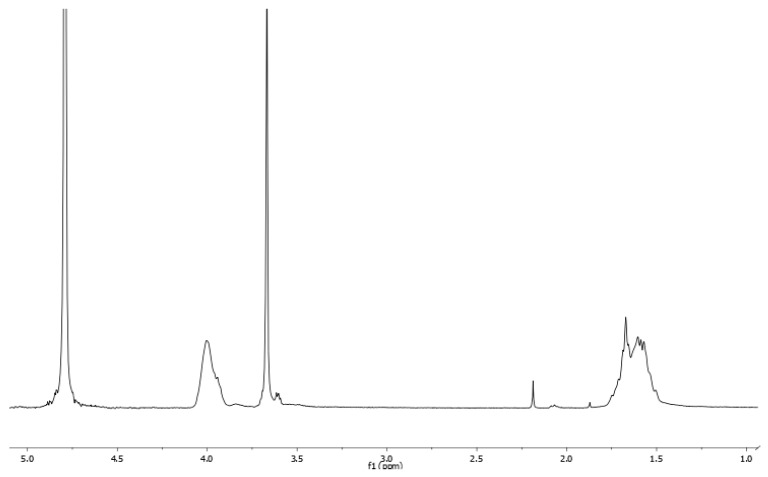
NMR Spectra of 83.32% PVA, 16.68% PEG.

A representative NMR spectrum for the highest PVA content of manufactured hydrogels is shown in Figure 1; characteristic methylene (CH_2_) and methine (CH) peaks can be seen for PVA around 1.6 ppm and 3.9 ppm, respectively, matching prior literature findings [27,28]. A characteristic methylene peak at 3.7 ppm can also be seen for PEG that others have reported [29]. By integrating the area of the methylene peaks for both PVA and PEG and accounting for the number of hydrogens at each peak location, the theoretical concentrations of dry mass can be determined in each hydrogel formulation. Spectra between samples with and without PVP were found to be identical and did not exhibit formation of a characteristic prominent signal from the CH_2_ groups in the pyrrolidone ring around 2.4–2.5 ppm that others have identified [30,31].

**Figure 2:**
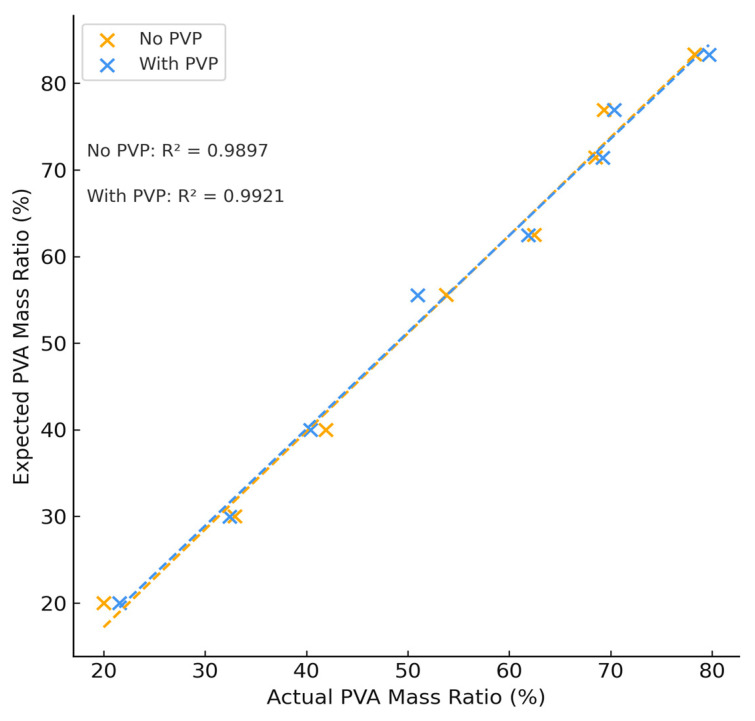
Theoretical vs. Actual PVA Concentration Due to NMR Testing.

The theoretical and calculated mass percent ratio of dry PVA content to PEG content of each hydrogel formulation is summarized in Figure 2 and agrees with expected values (see Table S1). The average difference between expected and theoretical values was found to be (2.77 ± 2.24)% and showed a strong linear correlation between expected and actual values of PVA with and without PVA, confirming the use of NMR spectroscopy as a method of quantification in multi-phase polymer systems.

### 3.2. APC

**Figure 3:**
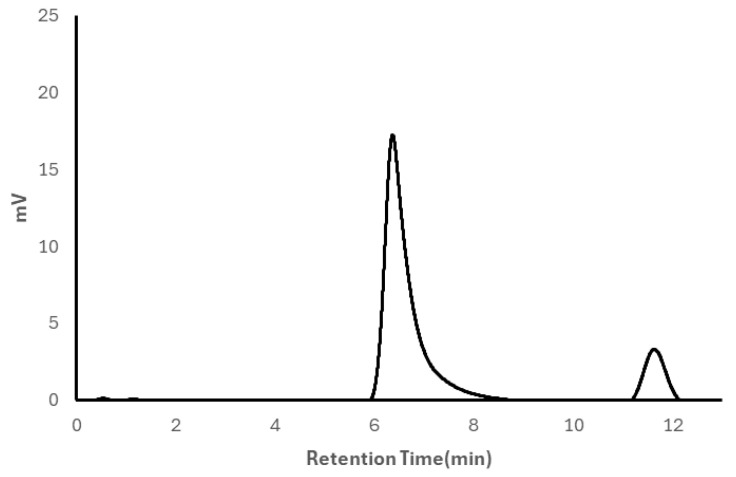
Representative APC Chromatogram of 83.32% PVA, 16.68% PEG.

A representative APC chromatogram for the highest PVA content of manufactured hydrogels is shown in Figure 3. In all collected chromatograms, retention time was independent of sample concentration and the presence or absence of PVP (see Figure S5). The peak for PVA reaches a maximum at 6.4 min. The peak for PEG is narrower than that of PVA and reaches a maximum at 11.8 min

**Figure 4:**
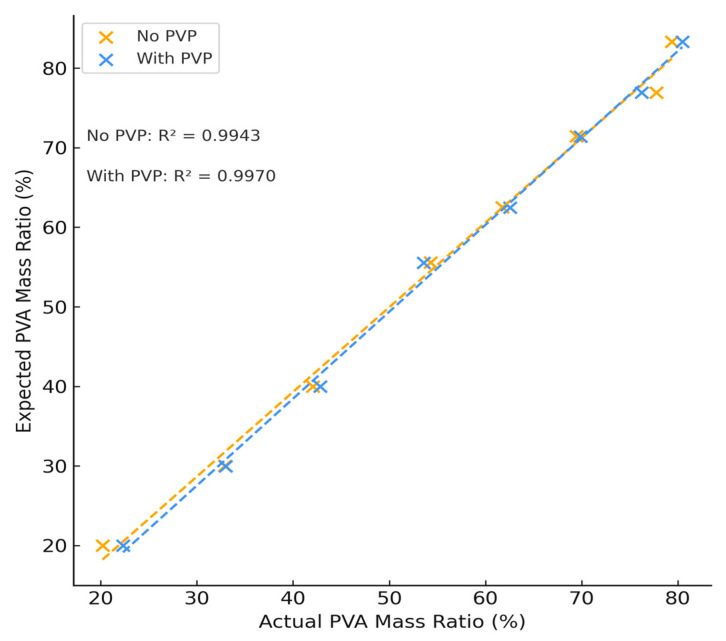
Theoretical vs. Actual PVA Concentration Determined by APC Testing.

Variations in PVA and PEG content resulted in a complementary increase or decrease in each respective peak. After integrating the area of these peaks, the concentration of PVA and PEG was calculated according to the concentration curves for each individual component and the mass percent of PVA of each hydrogel formulation is summarized in Figure 4 which shows a strong linear correlation between samples with and without PVP. The difference of this mass percentage can be used to calculate the mass percent of PEG. The calculated concentration agrees with expected values and the sample error of all samples tested was no greater than 4% (see Table S2). The average difference between expected and theoretical values was found to be (2.01 ± 1.19)%, performing slightly better than NMR and confirming the use of APC as an analytical method of polymer quantification for complex polymer systems.

### 3.3. PVA/PEG Hydrogel Component Analysis Through the Use of TGA

**Figure 5:**
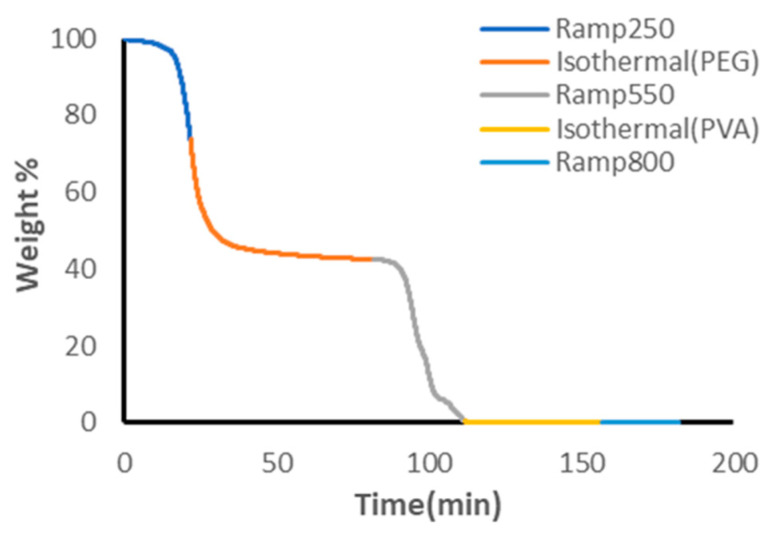
TGA Curve of PVA/PVP 99:1 *w*/*w*% and Non-Crosslinked PEG.

Figure 5 shows the TGA decomposition pattern of a stock solution of PVA/PVP (99:1 *w*/*w*%) and non-crosslinked PEG. Arbitrary mass values of 7 g of PVA and 8.5 g of PEG were used corresponding to theoretical values of 45.16% PVA and 54.84% PEG if PVP content is assumed negligible. The distinct plateaus in the isothermal heating cycle of PEG show that Equations (1) and (2) can be used to determine the independent weight percents of PVA and PEG which were found to be 42.39% and 57.61%, respectively. Theoretical values were comparable to experimental values for both PVA and PEG, validating the use of TGA as a method of polymer quantification.

**Figure 6:**
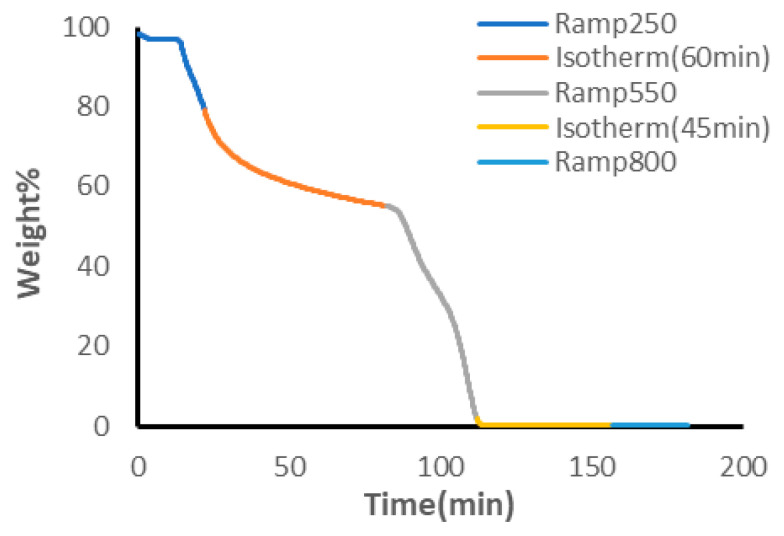
TGA Curve of 83.32% PVA and 16.68% PEG.

**Figure 7:**
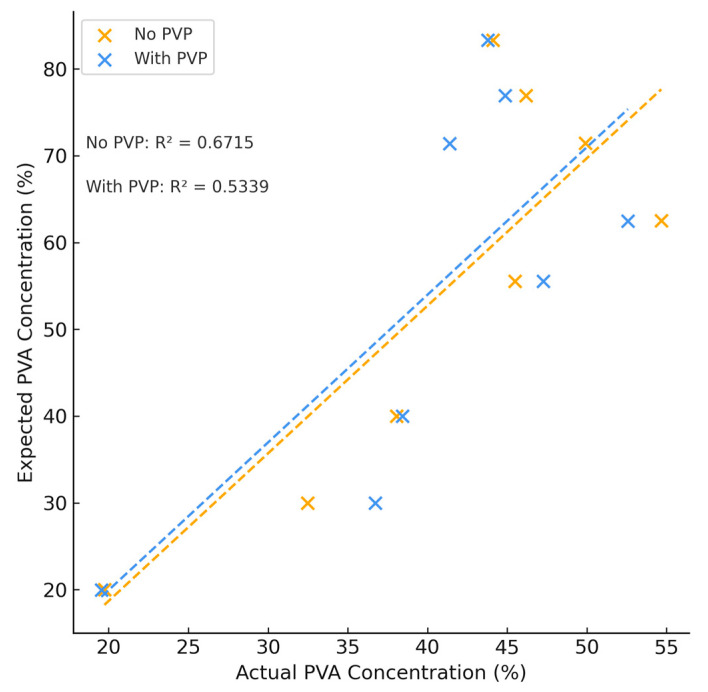
Theoretical vs. Actual PVA Concentration Due to TGA Testing.

Changes in the composition of PVA and PEG were identified in the TGA curves across samples tested. Figure 6 depicts the representative TGA curve for the highest PVA content of manufactured hydrogels. There were no observable changes noted between hydrogels made with or without PVP. In contrast to the curve in Figure 5, PEG did not produce a plateau across the isothermal heating stage at 250 °C, possibly indicating intermediaries or intermolecular bonding interactions that altered the decomposition patterns. When PVA concentrations were below 62.5%, the calculated concentrations of PVA agreed with expected values and the average difference between theoretical and expected values was found to be (4.95 ± 3.97)%. The error was less than or equal to 10% in all hydrogel formulations tested (see Table S3). The expected values of PVA, however, did not agree with those calculated from the TGA thermograms and the error was large when PVA concentrations exceeded 62.5% (see Figure 7). When the concentration was above this range, the calculated value of PVA was underestimated in all instances.

### 3.4. FTIR

**Figure 8:**
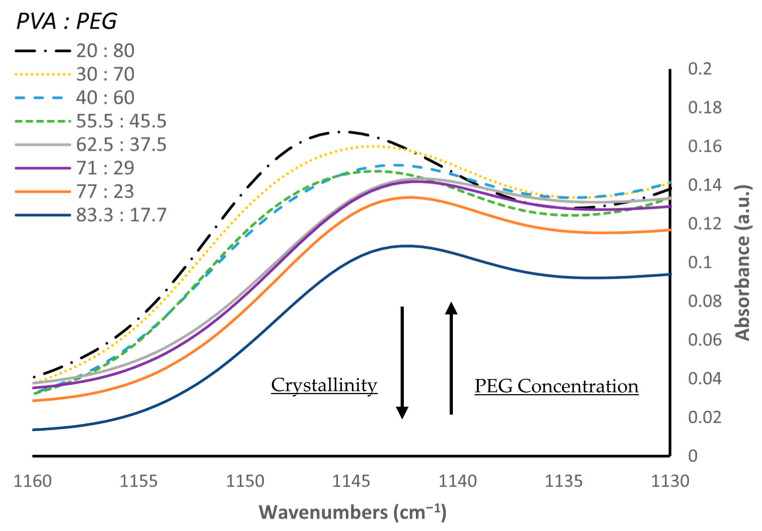
Average FTIR Spectra (PVA/PEG *w*/*w*%).

Figure 8 shows the FTIR region around 1140 cm^−1^, which others have reported to correlate with PVA crystalline formation [32]. The peak centered at 1146 cm^−1^ presents a maximum at the lowest PVA concentration (20% PVA) and decreases gradually with a corresponding increase in PVA content. A shift towards lower wavenumbers of 1142 cm^−1^ is identified as the concentration of PVA begins to exceed 62.5 *w*/*w*%. The spectra changes shape at this point and the intensity of this peak continues to decrease until it reaches a minimum when PVA concentration is highest at 83.3 *w*/*w*%.

**Figure 9:**
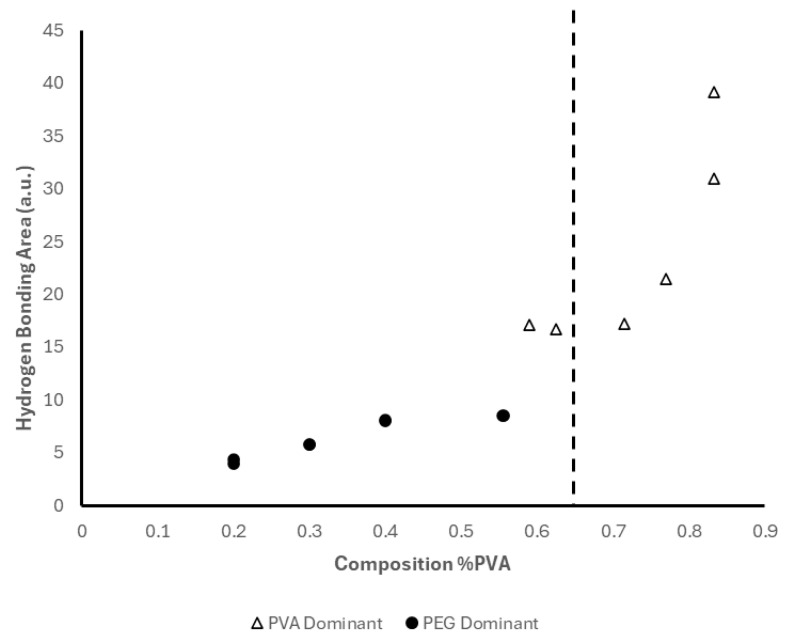
Area of the -OH Region of FTIR Spectra.

Our results are consistent with previous reports that a low-molecular weight PEG promotes the crystallization of PVA [33,34] and increasing PEG concentration lowers the degree of crystallinity, as evidenced by the shift in wavenumbers of the peak around 1140 cm^−1^. The shift in wavenumbers at PVA concentrations greater than 62.5% suggests a conformational change in the crystalline structure of the hydrogel. It is suggested that at higher PVA concentrations, the plasticizing effect of PEG is limited, allowing for larger crystalline structures to form between PVA and water. We also determined the area of the -OH bonding region between 3650 cm^−1^ and 3000 cm^−1^ which is summarized in Figure 9. The area of this region increases gradually for all samples in which PVA content is less than or equal to 55.5 *w*/*w*%. As this threshold is reached, the area increases sharply at 62.5 *w*/*w*% PVA and this marked increase is observed until its maximum at 83.3 *w*/*w*% PVA. The increase in the -OH bonding region further supports the crystalline conformational change between PVA and water observed by the shift in wavenumbers.

## 4. Discussion

NMR is widely recognized as a gold-standard method for determining polymer composition in multicomponent systems [35,36,37], and it has been used extensively to quantify the relative contributions of PVA, PEG, and related polymers in hydrogel formulations [38,39,40]. Here, NMR proved to be an effective tool to determine the polymer composition of both PVA and PEG across the different formulations of multi-component gels analyzed. NMR had a low average error of 2.77% and an R^2^ value of 0.99 across all samples tested, proving to be impervious to physiochemical interactions between polymers as nearly all polymers have unique protons for analysis [19,20]. APC, while having demonstrated use in multi-component polymer composition characterization, has yet to be evaluated as extensively in hydrogel formulations. Here, it was demonstrated that APC had a similar effectiveness in determining multi-component polymer content, showing a low error between expected and theoretical values of PVA and PEG across all samples tested (2.01%) and an R^2^ value of 0.99. Results were independent of physiochemical interactions within the hydrogel system and APC presented a higher-throughput method of analyzing polymer content when compared to NMR. Issues may arise, however, with overlapping peaks if the multi-component polymer system being analyzed has polymer components with similar molecular weights. Peak deconvolution or multi-detector coupling may be necessary to accurately determine individual polymer content of a complex polymer matrix system.

TGA, a method classically employed for understanding the thermal stability and decomposition patterns of polymers [41], similarly showed promise in characterizing the individual polymer content of our differing hydrogel formulations. At lower PVA concentrations below 62.5%, hydrogen bonding between PVA and PEG was low, allowing for quantification of individual polymer content as PVA and PEG exhibited their own unique thermal decomposition patterns with minimal contribution of inter-polymer physiochemical effects. However, as PVA content increased past this threshold, quantification was challenging and the R^2^ value was between 0.53 and 0.67 for samples with and without PVP, respectively. Hydrogen bonding also increased as evidenced by the sharp increase of the area of this region due to FTIR analysis. This increase in hydrogen bonding suggests the formation of additional microcrystalline structures between PVA and water. Furthermore, FTIR analysis revealed a shift in the wavenumbers of the crystalline region of PVA, suggesting a conformational change that influenced the thermal properties of the hydrogel blend that made individual polymer content challenging through the use of TGA.

At low PVA concentrations, TGA has the potential to serve as a method of characterizing individual polymer content for multi-component PVA-based blends, but a complete understanding of the physiochemical interactions between all components must be evaluated before analysis. Other researchers have shown the dependence of PVA melting rate on copolymers and found that the addition of catechin in a PVA transdermal delivery device promoted increased thermal stability [42]. Freeze-thaw cycles may also disrupt TGA analysis as it has been shown that multiple cycles increase crystallinity, especially at PVA concentrations greater than 10% [43]. Concurrently, variable amounts of PVP in a PVA-based hydrogel blend may further introduce TGA error. Others have shown that PVP concentration significantly influences the effectiveness of freeze–thaw cycling on hydrogel formation [44,45,46]. In PVA/PVP blends containing 1% PVP, the (OH) band shifted rapidly to higher wavenumbers after a single freeze-thaw cycle compared to pure PVA hydrogels formed under identical conditions, reaching a plateau as the number of freeze-thaw cycles increased to 20 in PVA hydrogels alone. In contrast, higher PVP concentrations (5–10%) combined with increased freeze-thaw cycles (e.g., 9 cycles) produced hydrogels dominated by stronger intermolecular hydrogen bonding (O–H···O–H), evidenced by a pronounced shift to lower wavenumbers. Notably, the authors reported that the (C–O–C) band at 1142 cm^−1^, indicative of crystallinity, remained unchanged across all formulations and freeze-thaw conditions, indicating that this vibrational mode is insensitive to both PVP content and freeze–thaw cycling [46]. This report suggests that the conformational crystallinity change we identified through FTIR is also independent of PVP content and number of freeze-thaw cycles, but rather a function of PVA/PEG ratio. APC, however, still presents as a viable alternative to individual polymer quantification of PVA-based hybrid hydrogels as it is independent of physiochemical interactions between polymer blends.

## 5. Conclusions

NMR and APC both served as effective methods to evaluate the individual polymer concentrations of PVA and PEG in our hybrid hydrogel formulations. Furthermore, these methods can be applied to other hydrophilic polymer blends as these methods were independent of the physiochemical interactions within our multi-component system. APC may serve as a more viable option due to ease of sample preparation and throughput, but peak deconvolution or alternative column chemistries may be necessary. At lower PVA concentrations, we presented a method of TGA analysis that was able to effectively quantify the individual amount of PVA and PEG in our hydrogel blend. At higher concentrations of PVA, hydrogen bonding and larger crystalline formation between PVA and water was identified which altered the unique thermal decomposition patterns of our hydrogel. It may be necessary to impose a more robust, multi-stage heating cycle that takes into account the unique physiochemical and crystalline reactions occurring within the hydrogel.

## Figures and Tables

**Table 1:** Formulations for the prepared PVA/PVP/PEG Blends.

	PVA Content (wt%)	PEG Content (wt%)
**With PVP**	83.32	16.68
	76.91	23.09
	71.41	28.59
	62.48	37.52
	55.53	44.47
	39.98	60.02
	29.98	70.02
	19.98	80.02
**No PVP**	83.33	16.67
	76.92	23.08
	71.43	28.57
	62.5	37.5
	55.56	44.44
	40	60
	30	70
	20	80

(Dry Mass Ratios for PVA/PVP/PEG Blends—PVP content is assumed negligible and PVA content is rounded up 0.01% in samples with “No PVP” in accordance).

## Data Availability

The original contributions presented in this study are included in the article. Further inquiries can be directed to the corresponding author.

## Supplementary Materials

The following supporting information can be downloaded at: https://www.mdpi.com/article/10.3390/polym18010048/s1, Figure S1: AUC of Different Concentrations of PVA; Figure S2: AUC of Different Concentrations of PEG; Figure S3: TGA Curve of PEG; Figure S4: TGA Curve of PVA; Figure S5: Representative APC Chromatogram of 83.32% PVA, 16.68% PEG (Right, w/PVP) and 83.33% PVA, 16.67% PEG (Left, No PVP); Table S1: Theoretical and Calculated Values of PVA Mass % Due to NMR Analysis; Table S2: Theoretical and Calculated Values of PVA Mass % Due to APC Analysis; Table S3: Theoretical and Calculated Values of PVA Mass % Due to TGA Analysis.

## Abbreviations

The following abbreviations are used in this manuscript:

PVA	Polyvinyl Alcohol
PEG	Polyethylene Glycol
PVP	Polyvinylpyrrolidone
NMR	Nuclear Magnetic Resonance
APC	Advanced Polymer Chemistry
FTIR	Fourier Transform Infrared Spectroscopy
TGA	Thermal Gravimetric Analysis
