# Supplementary material for: Evaluating Polymer Characterization Methods to Establish a Quantitative Method of Compositional Analysis Using a Polyvinyl Alcohol (PVA)/Polyethylene Glycol (PEG)—Based Hydrogel for Biomedical Applications
Source: Polymers (Basel). 2025 Dec 24;18(1):48. doi: 10.3390/polym18010048 (PMC12787944; doi:10.3390/polym18010048)
Supplement: Supplementary file 1 [file polymers-18-00048-s001.zip › polymers-4062010-supplementary.pdf]

Supplementary Materials

Figures S1 and S2 display the area under the curve (AUC) of different concentrations of PVA and PEG from 4mg/mL to 0.25mg/mL due to APC processing. A linear regression line was applied to each dataset and the slope of the line was used to determine the concentrations of PVA and PEG in all subsequent concentration measurements.

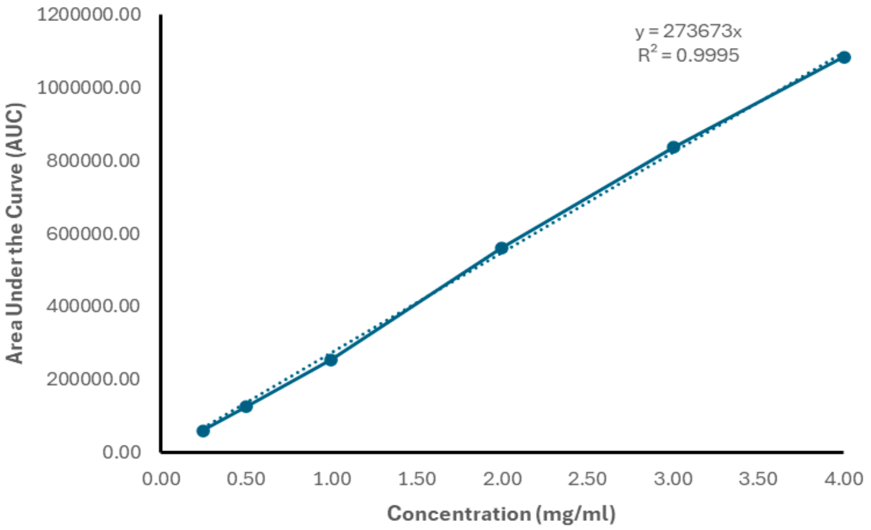

Figure S1. AUC of Different Concentrations of PVA

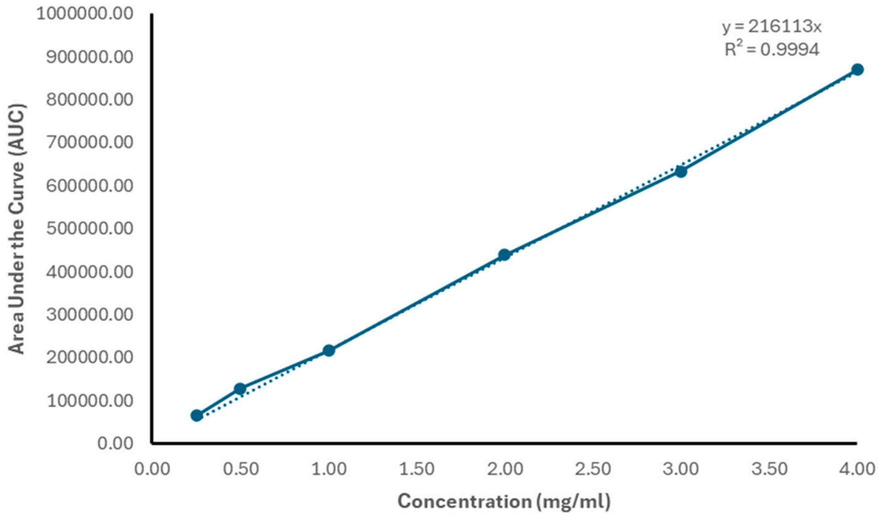

Figure S2. AUC of Different Concentrations of PEG

Table S1. Theoretical and Calculated Values of PVA Mass % Due to NMR Analysis

	Expected PVA Content (wt%)	Actual PVA Content (wt%)	% Difference
**With PVP**	83.32%	79.69%	3.63%
	76.91%	70.34%	6.56%
	71.41%	69.22%	2.19%
	62.48%	61.86%	0.61%
	55.53%	50.96%	4.57%
	39.98%	40.38%	0.41%
	29.98%	32.44%	2.46%
	19.98%	21.56%	1.58%
**No PVP**	83.33%	78.29%	5.04%
	76.92%	69.35%	7.58%
	71.43%	68.48%	2.94%
	62.5%	62.44%	0.06%
	55.56%	53.77%	1.78%
	40%	41.91%	1.91%
	30%	32.92%	2.92%
	20%	20.00%	0.00%
Average ± Standard Deviation			**2.77 ± 2.24%**

Table S2. Theoretical and Calculated Values of PVA Mass % Due to APC Analysis

	Expected PVA Content (wt%)	Actual PVA Content (wt%)	% Difference
**With PVP**	83.32%	80.31%	3.01%
	76.91%	76.22%	0.68%
	71.41%	69.89%	1.52%
	62.48%	62.57%	0.09%
	55.53%	53.56%	1.97%
	39.98%	42.80%	2.82%
	29.98%	33.00%	3.02%
	19.98%	22.31%	2.33%
**No PVP**	83.33%	79.37%	3.96%
	76.92%	73.24%	3.68%
	71.43%	69.53%	1.89%
	62.5%	61.77%	0.73%
	55.56%	54.28%	1.28%
	40%	42.04%	2.04%
	30%	32.89%	2.89%
	20%	20.19%	0.19%
Average ± Standard Deviation			**2.01 ± 1.19%**

Table S3. Theoretical and Calculated Values of PVA Mass % Due to TGA Analysis

	**Expected PVA Content (wt%)**	**Actual PVA Content (wt%)**	**% Difference**
**With PVP**	83.32%	43.78%	39.54%
	76.91%	44.87%	32.04%
	71.41%	41.39%	30.02%
	62.48%	52.58%	9.89%
	55.53%	47.27%	8.26%
	39.98%	38.42%	1.56%
	29.98%	36.72%	6.74%
	19.98%	19.54%	0.44%
**No PVP**	83.33%	44.11%	39.22%
	76.92%	46.19%	30.73%
	71.43%	49.91%	21.52%
	62.5%	54.67%	7.83%
	55.56%	45.49%	10.07%
	40%	38.05%	1.95%
	30%	32.47%	2.47%
	20%	19.73%	0.27%
Average ± Standard Deviation			**15.16 ± 14.48%**

Figures S3 and S4 show the TGA decomposition patterns of PEG and PVA, respectively. Significant decomposition for PEG begins around 200°C, with complete decomposition at 250°C and before the temperature ramp at 300°C. PVA experiences minimal decomposition until 300°C, with significant initial decomposition around 350°C. Complete decomposition of PVA was observed at 550°C.

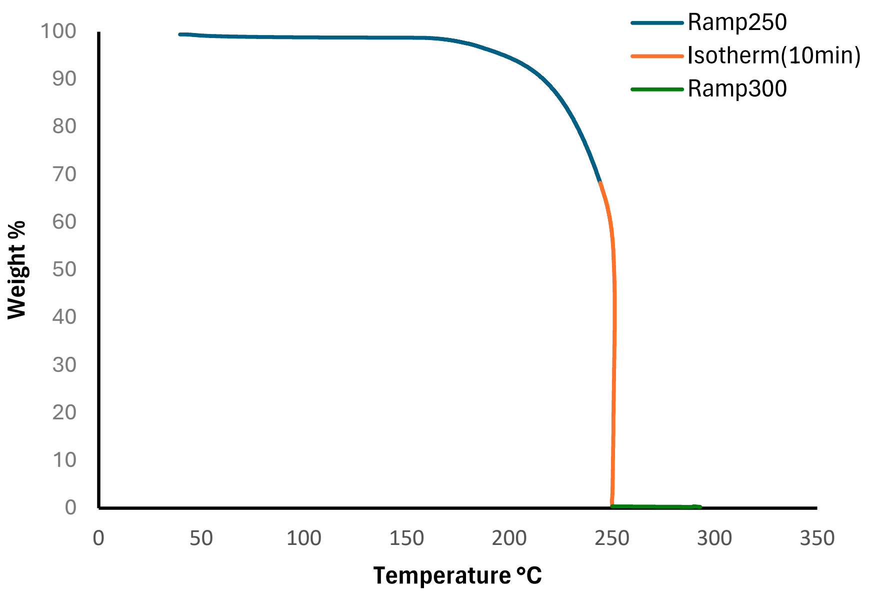

Figure S3. TGA Curve of PEG

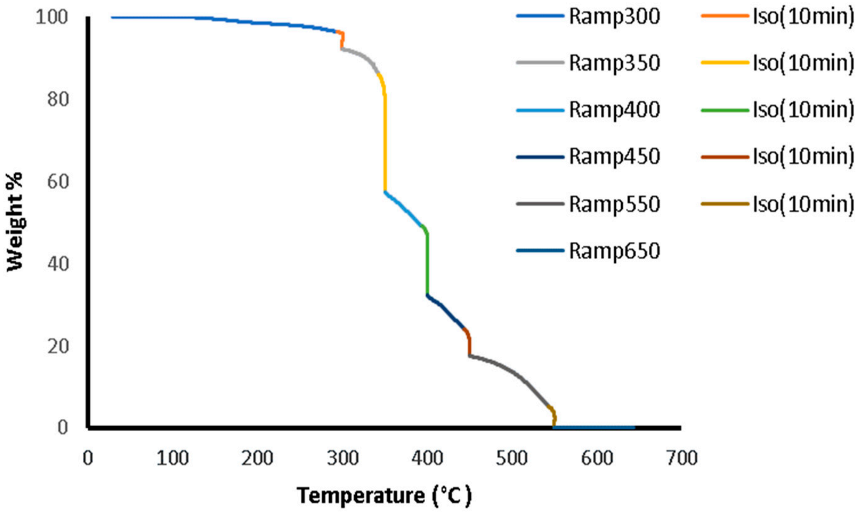

Figure S4. TGA Curve of PVA

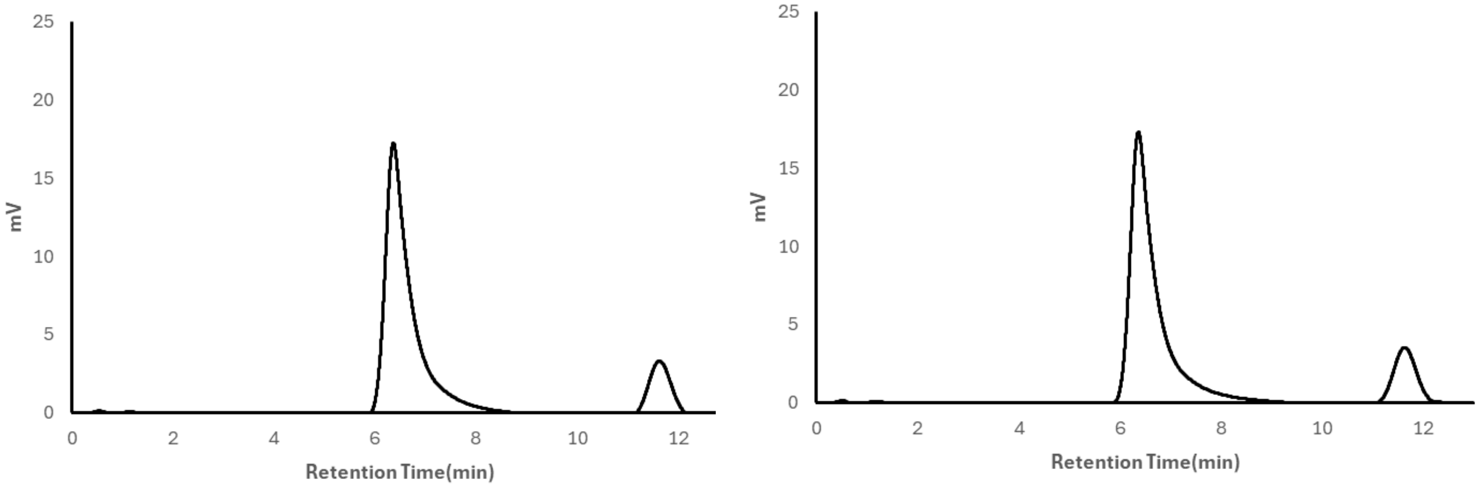

Figure S5. Representative APC Chromatogram of 83.32% PVA, 16.68% PEG (Right, w/PVP) and 83.33% PVA, 16.67% PEG (Left, No PVP)
